# Immunodetection of cells with a CD44+/CD24- phenotype in canine mammary neoplasms

**DOI:** 10.1186/1746-6148-9-205

**Published:** 2013-10-11

**Authors:** Geórgia Modé Magalhães, Erika Maria Terra, Rosemeri de Oliveira Vasconcelos, Márcio de Barros Bandarra, Pamela Rodrigues Reina Moreira, Mayara Caroline Rosolem, Antonio Carlos Alessi

**Affiliations:** 1 Faculdade de Ciências Agrárias e Veterinárias – FCAV/UNESP – Univ Estadual Paulista, Jaboticabal, Sao Paulo, Brazil; 2 Department of Clinics and Surgery, FCAV/UNESP-Jaboticabal, Address: Prof. Paulo DonatoCastellane, s/n, Jaboticabal, Sao Paulo 14884-900, Brazil

**Keywords:** Cancer stem cells, CD44, CD24, Canine neoplasia, Oncology, Mammary neoplasia, Canine, Stem cells

## Abstract

**Background:**

Cancer stem cells (CSCs) are able to self-renew and to form metastases. Using flow cytometry, CSCs were detected in canine mammary tumors as cells CD44^+^ and CD24^-^. The aim of this study was to detect these CSCs by immunohistochemistry and correlate their frequency with canine mammary neoplasm grade and histopathological type.

130 mammary neoplasm samples were selected from tissue blocks at the Department of Pathology at UNESP and classified according to (BJVP 4:153-180, [2011]). These samples were composed by adenomas, lymph node metastases, solid carcinomas grades II and III, tubular, papillary and carcinomas in mixed tumor grades I, II and III. Immunohistochemistry was performed with antibodies against CD44 and CD24. Linear regression was performed using Pearson’s correlation test.

**Results:**

The value at CD44 was positive and CD24 becomes zero was 46.75%. Cells with a CD44^+^/CD24^-^ phenotype were detected in 40 out of 130 samples with an advantage of high grade tumors (II and III) and metastases among tubular, papillary and carcinomas in mixed tumors. In these samples, percentages of cells stained by CD44 and CD24 antibodies were 62.2% and 0%, respectively. Published reports usually correlate grade III tumors with the expression of CD44 but not with CD24 expression. Studies using flow cytometry have found CSC frequencies similar to those found in our study.

**Conclusions:**

Immunohistochemistry was found to be a reliable technique for the detection of CSCs in canine mammary neoplasms, and the frequency of these cells positively correlates with grades II and III tumors (poor prognosis).

## Background

Stem cells have two major properties: the ability to self-renew by being able to divide and form another stem cell; and differentiation into new mature cells in the organ were they reside [1].

Cancer Stem Cells (CSC) have properties which are analogous to those of stem cells [2] and cannot be isolated and characterized as a simple cell. Specific cell surface markers for CSCs have been detected in human mammary neoplasms which stain positive for CD44 and negative or low for CD24. The discovery that these cell surface receptors can be used to define a CSC phenotype was made after the implantation of human solid mammary tumors in rats. The authors of that study demonstrated that solid tumors contained a small distinct population of cells with a unique ability to form tumors in non-obese, diabetic, immunosuppressed rats and these cells were named tumorigenic cells or cancer initiating cells [3].

For a better comprehension of these stem cells, the authors of one study [4] created an *in vitro* model in which CD44+/CD24- cells isolated by fluorescence-activated cell sorting form mammospheres [3]. Mammospheres are spherical colonies formed from one single non-adherent cultured cell which is capable of inducing the formation of tumors in rats.

CSCs have been observed in cultured cells derived from canine mammary neoplasms [5], although cells bearing self-renewal capacity were found to be rare. Cells from mammospheres formed from canine mammary gland cells were able to give rise to mammary ducts and alveoli *in vitro*[6]. In another study using mammospheres, CSCs in canine mammary neoplasms exhibited a CD44+/CD24- phenotype in four different tumoral cell lines [7].

In another study with canine cancer cell lines (osteosarcoma, melanoma, glioma and mammary tumor), CD44 expression was associated with proliferation, but the authors concluded that the transient and fluctuating expression may limit its utility as a CSC marker [8].

Abraham et al. [9] suggested that the CD44+/CD24- phenotype is not associated with clinical prognosis or survival time of human patients with mammary neoplasms, but is associated with presence of distant metastases, such as bone metastasis. Due to the high incidence of mammary neoplasms in female dogs, which ranges from 50% [10] to 68.4% [11] or 73.4% [12], immunodetection of CSCs can prove valuable for therapeutic choice and prognosis prediction in these patients. Thus, the aim of this study was to identify the CD44 and CD24 expressions in different canine mammary neoplasms, including benign, malignant and lymph node metastatic sites, and correlate them with histological grade of malignancy and histopatological type, since no similar data has been found in the literature.

## Methods

The present study was approved by the Ethics Committee on Animal Use (protocol number: 025600–08). A total of 130 mammary neoplasm samples (including benign, malignant and lymph node metastases specimens) were selected from the archives of the Veterinary Pathology Department (FCAV/Unesp - Jaboticabal).

Malignant neoplasms were composed of grades I, II, and III tubular, papillar, and carcinomas in mixed tumors and grades II, and III solid carcinomas. Adenomas were also selected. Lymph node metastases were randomly selected, however their primary tumor was not included, they were used as undifferentiated mammary neoplastic cells. All neoplasms were reclassified and graded according to the criteria proposed by [13], Table 1.

**Table 1:** Number of samples of canine mammary neoplasms distributed by histological type and grade

**Number of samples**	**Histological type and grade**
12	Grade I tubular carcinoma
12	Grade II tubular carcinoma
10	Grade III tubular carcinoma
10	Grade I papillary carcinoma
8	Grade II papillary carcinoma
8	Grade III papillary carcinoma
11	Grade II solid carcinoma
10	Grade III solid carcinoma
11	Grade I carcinoma in mixed tumor
11	Grade II carcinoma in mixed tumor
10	Grade III carcinoma in mixed tumor
8	Metastases
9	Adenomas

### Immunohistochemical analysis

For immunohistochemical analysis, serial tissue sections were made from paraffin-embedded mammary neoplasms and stained using antibodies against CD44, and CD24, as described in Table 2.

**Table 2:** Dilutions, clones and supplier of the antibodies used in canine mammary neoplasm tissue sections

**Antibodies**	**Clones**	**Dilutions**	**Supplier**
CD44, HCAM (M)*	IM7	1:100	Santa Cruz, ref. 18849
CD24 (M)	M1/69	1:75	Santa Cruz, ref. 19651

*M = Monoclonal antibody.

### Immunohistochemistry was performed using a polymer-based method

Formalin-fixed, paraffin-embedded tissue sections (4 μm thickness) of neoplasms were deparaffinized in xylene and rehydrated through graded concentrations of ethanol. After inhibition of endogenous peroxidase activity with 10% H_2_O_2_ (20 min), the antigen retrieval was achieved by heat treatment in a pressure cooker (Pascal, DAKO) in Tris-EDTA buffer at pH 9.0 for the CD24 antibody. For CD44 antibody, the antigen retrieval was carried out using citrate buffer pH 6.0 also in a pressure cooker.

After three thorough washes with Tris–HCl solution (pH 7.4) for 5 minutes, blocking for non-specific binding was performed using a blocking solution (protein block serum-free – DAKO ref. ×0909). Sections were incubated with antibodies at optimal dilution (Table 2) for 18 hours (*overnight)* at 4°C for CD24 and 1 hour at 28°C for CD44. Then, the sections were incubated with a peroxidase-conjugated polymer (kit ENVISION + Dual Link System Peroxidase ref K4061 - DAKO) and diaminobenzidine (DAB - DAKO, ref. K3466) was used as the chromogen. Hematoxylin was used as the counterstain.

The primary antibody was replaced with antibody diluent (Antibody Diluent with Background Reducing Components, ref. S3022, DAKO) as a negative control. Breast tumors tissue sections known to express these markers were used as positive controls in each batch of IHC analysis. The use of human material was approved by the Research Ethics Committee of CHRP and FMRP/USP (process 242/2011).

### Quantification of stained cells

The type and distribution of stained cells were analyzed before cell counts were performed. Four fields were randomly selected to determine the number of stained cells. A total of 100 stained or non-stained cells were counted using the 40× objective. Results were expressed in percentage of stained cells. In the carcinomas in mixed tumors group, only epithelial neoplastic cells were counted because there was no labeling in mesenchymal and myoepithelial cells. These epithelial cells correspond to carcinomatous (malignant) areas of this tumor.

### Statistical analysis

Data were analyzed using Pearson’s correlation test followed by Simple Linear Regression using SAS software (SAS 9.1, SAS Institute, Cary, NC, USA). The frequency of histopathological types was analyzed using the 95% confidence interval.

## Results

**Figure 1:**
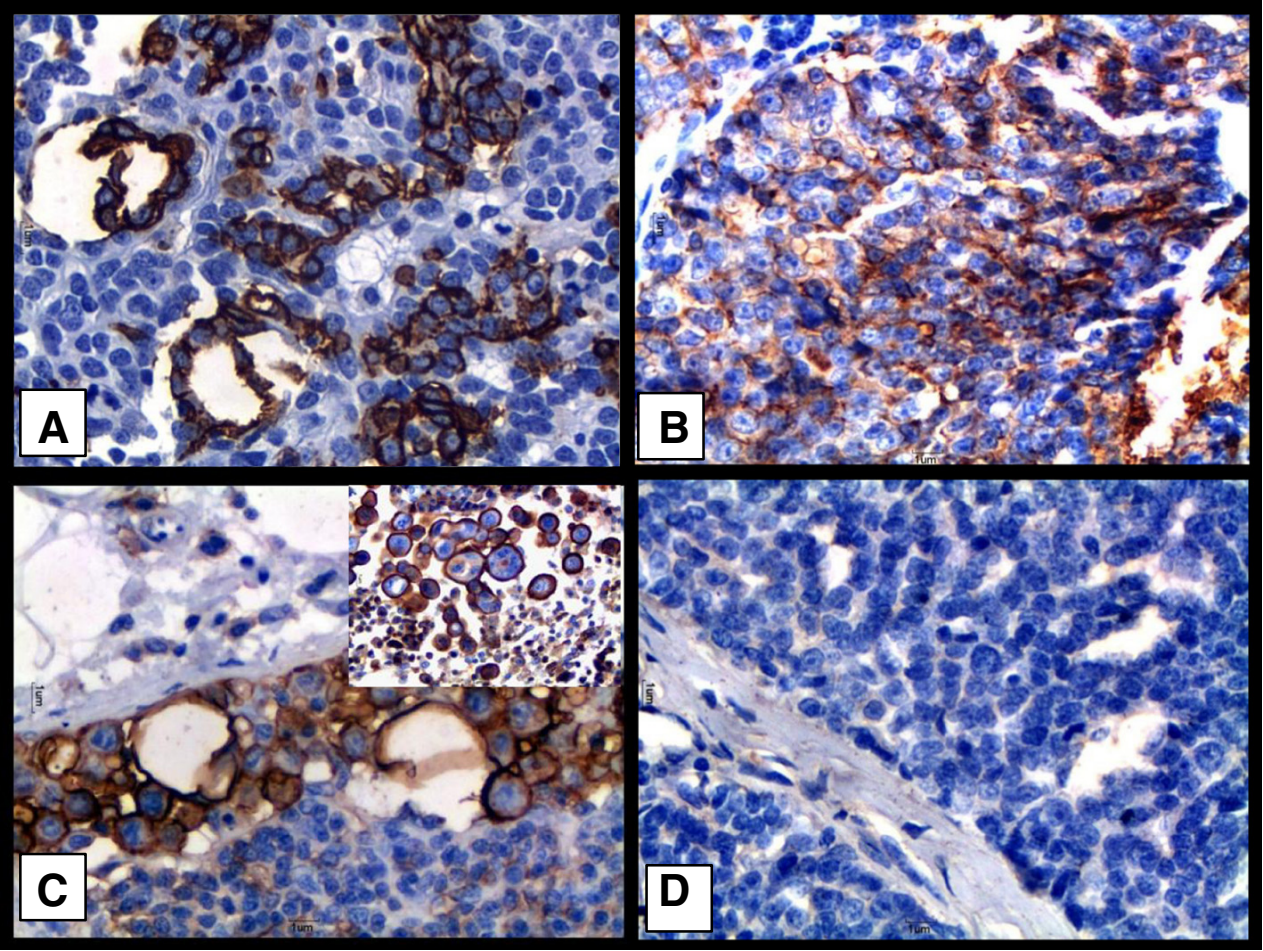
**Immunostaining of CD44 and CD24 indifferent types of canine mammary neoplasms.** Legend: **(A)** Grade II mixed tumor carcinoma; note positive immunostaining for CD44 on the plasma membranes of neoplastic epithelial cells. **(B)** Grade III solid carcinoma; note positive immunostaining for CD44 on the plasma membrane of neoplastic epithelial cells. **(C) **Positive immunostaining for CD44 on the plasma membrane of undifferentiated epithelial cells. **(D)** Grade III solid carcinoma from a female dog; note absence of immunostaining on epithelial cells subjected to immunohistochemistry for CD24. Sections stained with DAB and counterstained with Harris Hematoxylin. 40× obj.

**Figure 2:**
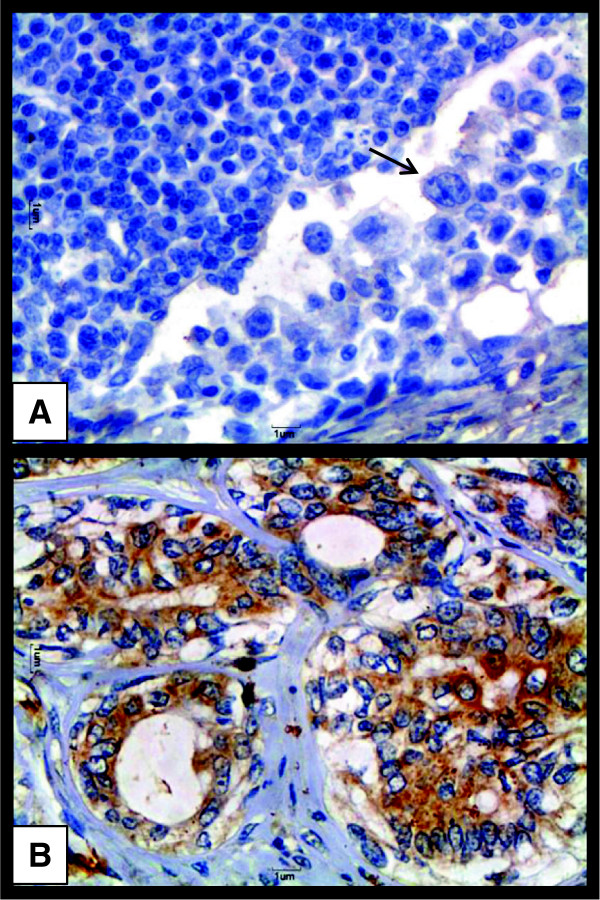
**CD24 Expressionin a canine malignant mammary neoplasm and a lymph node metastasis.** Legend: **(A)** Note absence of immunostaining for CD24 on metastatic cells (arrow) in a lymph node. 40× obj. **(B)** Grade I mixed tumor carcinoma from a female dog; note positive immunostaining for CD24 on the plasma membrane and in the cytoplasm of neoplastic epithelial cells. 40× obj.

Staining for CD44 was detected on the plasma membrane (Figure 1A). In solid carcinomas, staining was seen on myoepithelial cells and epithelial cells and was strongly positive (Figure 1B). Conversely, in carcinomas in mixed tumors, immunostaining for CD44 was seen on epithelial cells but not in well-differentiated mesenchymal tissue or in myoepithelial cells. Staining was strong on undifferentiated metastatic cells in samples from lymph node metastastic sites (Figure 1C). Staining for CD44 was more frequent in higher-grade tumors. Staining for CD24 was detected on the plasma membrane and in cytoplasm. In solid carcinomas, samples which stained positive for CD44 were negative for CD24 (Figure 1D). Metastatic cells in lymph nodes were likewise negative for CD24 (Figure 2A). In general, there were more positive cells for CD24 in grade I tumors (Figure 2B) compared to grade II and III tumors.

**Figure 3:**
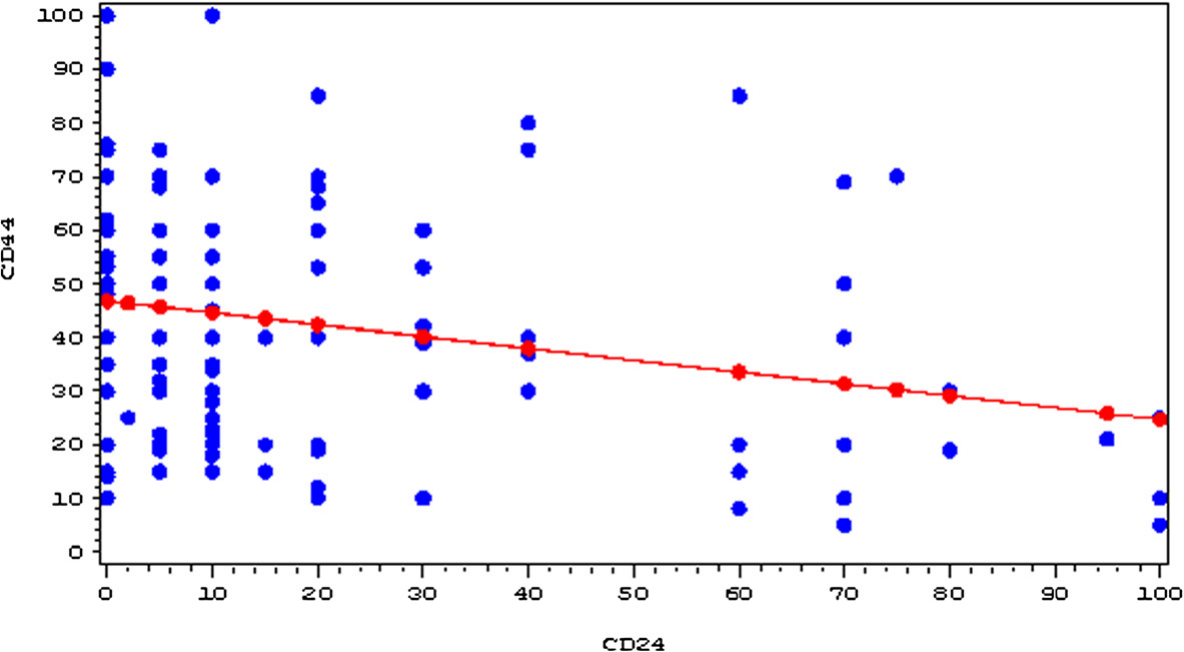
**Linear regression line between CD44 (y-axis) and CD24 (x-axis) using Pearson’s correlation test.** The value at which CD44 is positive and CD24 becomes zero is 46.75%.

Pearson’s correlation test performed on 130 samples marked with anti-CD44 and anti-CD24 antibodies exhibited a significant correlation with p = 0.0045. The intercept value of 46.75% ± 2.34 indicates that increased frequency of CD44+ cells correlates with decreased CD24+ cells. A regression line is shown in Figure 3.

Therefore, among the 130 samples analyzed, those that were positive for this correlation are represented in Table 3. From 130 samples, 40 (30%) expressed this phenotype (CD44+/CD24-); of these, 37 were high-grade tumors (II and III) and lymph node metastases and only three samples were grade I neoplasms. The mean number of cells which stained positive for CD44, and for CD24 in these samples was 62.2% and 0%, respectively. These neoplasms exhibited a CD44+/CD24- phenotype.

**Table 3:** Identification of canine mammary neoplasms expressing a CD44+/CD24- phenotype

**Neoplasm**	**Number of samples with a CD44+/CD24cp phenotype**
Grade I Tubular carcinoma	1 (2,5%)
Grade II Tubular carcinoma	5 (12,5%)
Grade III Tubular carcinoma	5 (12,5%)
Grade I Papillary carcinoma	1 (2,5%)
Grade II Papillary carcinoma	3 (7,5%)
Grade III Papillary carcinoma	4 (10%)
Grade II Solid carcinoma	1 (2,5%)
Grade III Solid carcinoma	2 (5%)
Grade I carcinoma in mixed tumor	1 (2,5%)
Grade II carcinoma in mixed tumor	4 (10%)
Grade III carcinoma in mixed tumor	6 (15%)
Metastases	7 (17,5%)
**Total**	40 (100%)

Lymph nodes metastatic sites represented the major frequency of positive cells for this phenotype (7/8; 87.5%; CI 95% 51.75-97.19%).

The most common histopathological type showing this phenotype was carcinoma in mixed tumors (11/32, 34.37%; CI 95% 36.21-57.76%), followed by tubular carcinomas (11/34; 32.35%; CI 95% 19.13-49.29%), papillary carcinomas (8/26; 30.77%; CI 95% 16.52-50.18%) and solid carcinomas (3/21, 14.29%; CI 95% 5.19-34.91%).

## Discussion

Other authors have detected a CD44+/CD24- phenotype in 91.9, 93.0, 76.2, and 44.3% of four different cell lines from canine mammary neoplasms using flow cytometry [7], however the immunohistochemical expression of these proteins was not demonstrated in canine mammary neoplasms. The percentages of cells bearing the CD44+/CD24- phenotype as assessed by immunohistochemistry in this study are very similar to those values for flow cytometry. Therefore, immunohistochemistry can be viewed as a good technique for the detection of CSCs in canine mammary neoplasms.

The percentage of cells which express this phenotype in human mammary neoplasms varies significantly. One study found a variation of 2% to 40% in the number CSCs detected by immunohistochemistry and, also, a high association of these cells with tumor grade and aggressiveness [14].

Of the 40 neoplasms with the CD44+/CD24- phenotype described in our study, 37 exhibited histological grades II or III, or were of lymph node metastases. Only three samples corresponded to grade I neoplasms. Other authors have found more CSCs in grade III (undifferentiated) than in grade I (well differentiated) human mammary neoplasms [15]. When these results were applied to the meta-analysis of breast cancer expression data sets, they found that it was predictive of biological and molecular features of human breast cancers and concluded that breast cancers can be distinguished based on their degree of resemblance to the CSCs molecular phenotype.

The most frequent histopathological type showing this CD44^+^/CD24^-^ was carcinoma in mixed tumors, followed by tubular, pappilary and solid carcinomas. In solid carcinomas, only three of 21 grade II or III mammary carcinomas evaluated in this study exhibited this phenotype.

Studies involving CSCs markers started with solid mammary tumors. Al-Hajj et al. [3] stated that solid tumors exhibited a distinct population of cells with a select ability to form tumors in rats and named them tumorigenic cells or cancer initiating cells because they were really able to give rise to new neoplasms. However, the isolation of CSCs in solid tumors has been difficult for several reasons. Cells from solid tumors are generally less accessible than normal stem cells in developing organs, and their detection and quantification require different functional assays as well [16]. In our study, the number of cells with a CD44+/CD24- phenotype was higher in the other histological types than in solid carcinomas, indicating that although this phenotype correlates with the worst histopathological grades, the prognostic association with histological type should be investigated.

Seven of 8 samples from lymph node metastases analyzed had the CD44+/CD24- phenotype. Other study has found similar, but not identical, results since the authors compared neoplastic cells in lymph nodes metastases with their corresponding primary tumor and found that metastatic cells contained a higher frequency of CD44^+^/CD24^-^ cells [17]. In our study, the metastatic lymph nodes were not evaluated with their primary tumors, but this fact is nonetheless likely to demonstrate that metastatic sites cells can have a higher population of cancer stem cells.

This CSC phenotype has also been detected in lymphovascular emboli of inflammatory breast carcinoma in women [18]. Another study using flow cytometry showed that 6.1% of the lymph node metastases contained CD44+/CD24- cells and found an association between metastatic dissemination and increased numbers of cells with this phenotype in human mammary neoplasms [19]. Some researchers believe that the cells that are able to migrate to metastatic sites are the CSCs [20]. There are so many pathways involved in metastatic process and one of the most important is the EMT (*epithelial-mesenchymal transition*) which is associated with a gain of stem cell-like behaviour. The most important steps for distant metastasis are dissemination through a fine net of blood vessels and colonization at the metastatic site. In contrast to undifferentiated, anaplastic primary tumours, cells from differentiated tumours are not expected to possess the necessary traits with which to disseminate, nevertheless they also metastasize [21], this way a correlation between cells in primary tumors and in their metastatic site is not always logic.

## Conclusion

The CD44+/CD24- phenotype can be detected by immunohistochemistry and is related to the most aggressive tumor grades in canine mammary neoplasms.

## Competing interests

The authors declare that they have no competing interests.

## Author’s contributions

GMM designed the study, carried out the lab analysis, and drafted the manuscript. EMT assisted in the preparation of the study, data collection and writing of the manuscript. Both GMM and EMT works equally in the study. ROV participated in the sequence alignment, assisted in imunoassays and revised the final version of the manuscript. MBB and PRRM carried out the immunoassays and performed the statistical analysis. ACA conceived the study, participated in its design and coordination, and helped to draft the manuscript. All authors read and approved the final manuscript.
